# Baseline Health and Nutritional Parameters of Wild Sand Tigers Sampled in Delaware Bay

**DOI:** 10.1002/aah.10156

**Published:** 2022-07-01

**Authors:** Lisa A. Hoopes, Tonya Clauss, Bradley M. Wetherbee, Dewayne A. Fox

**Affiliations:** ^1^ Georgia Aquarium, Department of Research and Conservation 225 Baker Street Northwest Atlanta Georgia 30313 USA; ^2^ Georgia Aquarium, Department of Animal and Environmental Health 225 Baker Street Northwest Atlanta Georgia 30313 USA; ^3^ Department of Biological Sciences University of Rhode Island 9 East Alumni Road Kingston Rhode Island 02881 USA; ^4^ Guy Harvey Research Institute Nova Southeastern University Dania Beach Florida 33004 USA; ^5^ Department of Agriculture and Natural Resources Delaware State University 1200 North Dupont Highway Dover Delaware 19901 USA

## Abstract

Species‐specific hematological reference values are essential for diagnosis and treatment of disease and maintaining overall health of animals. This information is lacking for many species of elasmobranchs maintained in zoos and aquaria, thus reducing the effectiveness of care for these animals. Descriptive statistics and reference intervals were calculated for hematocrit and complete blood cell counts, biochemistry and protein electrophoresis parameters, trace minerals, vitamins, heavy metals, reproductive hormones, and fatty acids in the blood of 153 wild Sand Tigers *Carcharias taurus* of both sexes and a range of sizes caught in Delaware Bay (Delaware, USA). Mean hematocrit, total white blood cell counts, lymphocyte differentials, glucose, phosphorus, amylase, and aspartate aminotransferase levels were significantly higher in juveniles than in adults. Levels of estradiol, progesterone, testosterone, and differences in selenium and eicosapentaenoic acid (a polyunsaturated fatty acid) between males and females suggest that they are important parameters for improving Sand Tiger breeding success in managed care. Finally, blood metal levels for arsenic, cadmium, lead, and mercury suggest low levels of contaminant exposure for Sand Tigers during their summer residence in Delaware Bay. The results of this study provide baseline health parameters for wild Sand Tigers that will aid in effective maintenance of aquarium animals and contribute to a greater understanding of the biology of these sharks and efforts to accomplish sustainable management of their populations.

Species‐specific hematological parameters are important tools for evaluating and understanding health and nutrition in both wild and managed populations of animals. Diet, environmental features, activity level, social structure, stress, and parasite loads are all factors that may influence blood values in zoo and aquarium animal collections (Ahmed et al. 2020). Population‐based reference intervals (RIs) for blood analytes are essential for the clinical diagnosis and treatment of disease in fish health; however, this information is lacking for many aquarium species (Otway et al. 2011; Ahmed et al. 2020). Despite the large diversity of elasmobranchs in zoos and aquaria, hematological RIs have been established for only a small number of species (Harms et al. 2002; Cain et al. 2004; Ferreira et al. 2010; Otway et al. 2011; Otway 2015; Cusack et al. 2016), and these results typically have small sample sizes and/or a limited panel of analytes.

The Sand Tiger *Carcharias taurus* is one of the most popular elasmobranch species displayed in public aquaria worldwide (Anderson et al. 2012) due to its large size, hardiness, and impressive dentition. However, aquarium‐held Sand Tigers frequently experience health issues that appear to be uncommon in the wild. For example, nearly one‐third of all Sand Tigers displayed in aquaria have developed spinal deformities, which include spinal curvature, kyphosis, single to multiple incremental subluxations, excessive mineralization of the vertebrae, and/or spinal degeneration (Anderson et al. 2012). Various explanations have been postulated for these spinal deformities, including capture and transport trauma, high growth rates, infection, and nutritional deficiencies (Preziosi et al. 2006; Anderson et al. 2012). Conclusive identification of the causes of spinal deformity have been elusive, partly due to a lack of complementary information for wild Sand Tigers.

Global Sand Tiger stocks have experienced dramatic declines; since they have one of the lowest rates of reproduction among elasmobranchs, the full recovery of stocks is expected to require decades (Carlson et al. 2009; Pollard and Smith 2009; Kilfoil et al. 2017). Concern about the negative effects of collecting wild Sand Tigers has also generated keen interest in focused breeding of Sand Tigers in aquaria (Henningsen et al. 2017). Although a variety of reproductive behaviors has been observed in aquarium Sand Tigers (Gordon 1993), successful reproduction under managed care has been extremely limited (Henningsen et al. 2017; Wilson and Smith 2017). This lack of success is likely related to an inability of aquaria to effectively mimic wild environmental conditions, behavioral cues, and/or physiological responses of Sand Tigers to artificial conditions that interfere with reproduction. Understanding the range of hematological values found among wild Sand Tigers is therefore likely to enhance efforts toward successful breeding by providing reference values for wild and aquarium animals.

The objectives of this study were to (1) provide a comprehensive suite of hematological, biochemical, and nutritional values (complete blood counts, plasma biochemistry, protein electrophoresis, trace minerals, vitamins, heavy metals, hormones, and fatty acids) for wild Sand Tigers in Delaware Bay; (2) compare blood analytes between sexes and among demographic groups of wild individuals; (3) establish RIs for wild Sand Tigers for comparison with aquarium individuals; and (4) infer the overall health of individuals collected in Delaware Bay based on hematological parameters.

## Methods

### Animal collection

Sand Tigers were caught during August 2011 (*N* = 60) and 2012 (*N* = 93) in Delaware Bay (39°03′00″N, 75°08′59″W) over 15 total days of effort using anchored longlines consisting of 366‐m, 0.64‐cm braided nylon, with 25 (16/0) circle hooks (barbs depressed) spaced 12 m apart. Hooks were baited with Atlantic Menhaden *Brevoortia tyrannus* or Bluefish *Pomatomus saltatrix*. Longlines were fished during daylight hours using soak times of approximately 2 h. Based on the time required for deployment and retrieval of the longline, however, some individuals could have been on hooks for periods exceeding 2 h. For each longline set, location (determined by GPS), surface water temperature, salinity, and dissolved oxygen were recorded.

Sharks were either immediately brought on deck (<150 cm TL) or secured adjacent to the boat with a tail rope (>150 cm TL). Animals were then rolled into dorsal or lateral recumbency, inducing a natural and temporary state of inactivity (tonic immobility), for workup. Individuals were measured (FL and TL), sexed, and tagged externally with conventional “spaghetti” identification tags; a subset of sharks received an internal acoustic transmitter for a separate study. Twelve milliliters of blood were collected (<1% of total blood volume) via caudal venipuncture using 18–21‐gauge needles of varying length (dependent on animal size) attached to a syringe via an extension set. For sharks that were processed onboard, the gills were irrigated with salt water via a hose inserted in the mouth and the skin was kept wet during handling. Total handling time for both onboard and in‐water processing was <15 min.

### Hematology and cell counts

Whole blood was immediately transferred into a 1‐mL sodium citrate blood tube, a 10‐mL lithium heparin blood tube, and an i‐STAT cartridge (Abaxis, Union City, California) for blood gas analysis (see below). Sodium citrate whole blood was added to a cryovial containing a modified Natt–Herrick's stain (Vetlab Supply, Palmetto Bay, Florida) at a 1:100 dilution for total white blood cell (WBC) counts to be conducted at a later time. Natt–Herrick's stain was modified for elasmobranch osmolality (1,190 milliosmoles/L) by mixing 25 μL of Natt–Herrick's stain with 0.79 g of urea and 0.34 g of sodium chloride (Walsh and Luer 2004; Arnold 2005). Heparinized whole blood (0.5 mL) was immediately aliquoted for heavy metal testing and held frozen at −80°C until analyzed. The remainder of the blood was held on ice until centrifuged (1,534 × *g* for 10 min), approximately 60–120 min after collection, and plasma samples were stored frozen at −80°C until analyzed.

Between 2 and 6 h postcollection, two blood smears were made for a WBC differential count using sodium citrate‐treated whole blood that had been kept chilled at 4°C. The blood smears were allowed to air dry and then were stained with Diff Quick (Dip Quick Stain Set; Jorgenson Labs, Loveland, Colorado). Blood smears were examined using light microscopy (1,000× magnification), and the differential count was read twice by identifying 100 individual WBCs and calculating the percentage of each cell type. White blood cells were classified as lymphocytes, neutrophils, basophils, monocytes, coarse eosinophilic granulocytes (CEGs), and fine eosinophilic granulocytes (FEGs) using criteria defined by Arnold (2005). Segmented and nonsegmented CEGs and FEGs were differentiated since they represent differing stages of cell maturity. A manual total WBC count was performed on Natt–Herrick's stained whole blood by using a hemacytometer (Walsh and Luer 2004). Hematocrit was measured by microhematocrit centrifugation, and total solids were measured with a refractometer (HSK‐VET; Heska Corporation, Loveland, Colorado).

### Acid–base and blood gases

Blood gas analyses were conducted using an i‐STAT portable clinical analyzer (Abaxis) and a CG4+ disposable cartridge that measured pH, partial pressure of carbon dioxide (pCO_2_; mm Hg), partial pressure of oxygen (pO_2_; mm Hg), lactate (mmol/L), bicarbonate (HCO_3_; mmol/L), total carbon dioxide (mmol/L), and percent oxygen saturation (sO_2_; %). Since mammalian conversion factors and constants are utilized by the i‐STAT to make temperature corrections to pH, pCO_2_, and pO_2_, water temperature was used as a proxy for body temperature so that values could be of diagnostic use in elasmobranchs (see Mandelman and Skomal 2009).

### Biochemistry profiles

Plasma biochemical profiles were performed at Michigan State University's (MSU) Diagnostic Center for Population and Animal Health (DCPAH) using an Olympus AU640e chemistry analyzer (Olympus Diagnostics, Melville, New York). This profile included blood urea nitrogen (BUN), creatinine, sodium, potassium, chloride, calcium, phosphorus, magnesium, iron, glucose, amylase (enzyme number 3.2.1.2; IUBMB 1992), alkaline phosphatase (ALP; 3.1.3.1), alanine aminotransferase (ALT; 2.6.1.2), aspartate aminotransferase (AST; 2.6.1.1), creatinine phosphokinase (CPK; 2.7.3.2), and cholesterol. Reported osmolarity values were calculated based on (2 × Na^+^) + (glucose/18.0) + (BUN/2.8).

### Protein electrophoresis

Plasma protein fractions were evaluated by an electrophoresis analysis system and gels (SPIFE 3000 system, Split Beta gels; Helene Laboratories, Beaumont, Texas) at the University of Miami's Avian and Wildlife Laboratory according to the manufacturer's instructions and as previously described (Cray et al. 2011). Protein fractions were based on mammalian electrophoresis migration characteristics for albumin, α‐1‐globulin, α‐2‐globulin, β‐globulin, and γ‐globulin but were identified as fractions 1–5 (Cray et al. 2015). Absolute fraction values (g/dL) were determined by multiplying the percentages for each fraction by the total protein (TP) concentrations measured with an Olympus AU640e chemistry analyzer.

### Reproductive hormones

Plasma hormone levels (estradiol, progesterone, and testosterone) were measured using solid‐phase ^125^I radioimmunoassay at Cornell University's Animal Health Diagnostic Center. Prior to being assayed, estradiol samples were extracted with ethyl ether, and titrated ^3^H‐estradiol was used for determining percent recovery of each sample extracted and final calculations. Standard curves were prepared by the laboratory and by using Siemens Coat‐A‐Count Estradiol antibody‐coated polypropylene tubes and tracer (Siemens Medical Solutions Diagnostics, Los Angeles, California). Similarly, for the quantification of progesterone and testosterone, Siemens Coat‐A‐Count reagents were used, which included calibrators with different levels of hormone, antibody‐coated polypropylene tubes, and ^125^I‐labeled progesterone or testosterone.

### Trace minerals and heavy metals

Trace mineral and heavy metal analysis was based on the methods described by Wahlen et al. (2005). Plasma and whole‐blood samples were diluted 20‐fold with a solution containing 0.5% EDTA and Triton X‐100, 1% ammonium hydroxide, 2% propanol, and 20 ng/mL of scandium, rhodium, indium, and bismuth as internal standards. Due to the viscous state after the initial dilution, blood samples were additionally diluted 5× in Millipore water and digested with 3 μL of ultrapure 30% hydrogen peroxide for a minimum of 20 min. Whole‐blood samples were analyzed for arsenic (As), cadmium (Cd), lead (Pb), mercury (Hg), selenium (Se), and thallium (Tl); plasma samples were analyzed for cobalt (Co), copper (Cu), manganese (Mn), molybdenum (Mo), zinc (Zn), and Se using an Agilent 7500ce inductively coupled plasma mass spectrometer (Agilent Technologies, Santa Clara, California). Elemental concentrations were calibrated using a four‐point linear curve of the analyte–internal standard response ratio.

### Vitamins

Plasma vitamin A (retinol) and vitamin E (α‐tocopherol) levels were analyzed by MSU‐DCPAH using the methods described by Arnaud et al. (1991). Fat‐soluble vitamins were extracted with equal volumes of ethanol (which included an internal standard and butylated hydroxytoluene) and hexane. The hexane layer was dried under reduced pressure and dissolved in chromatographic mobile phase. Samples were analyzed chromatographically using a Waters 2690 Alliance separation module and Waters 996 photodiode array detector (Waters Corporation, Milford, Massachusetts). Quantification was by internal standard ratios and a six‐point calibration curve. Calibration standard solutions were verified by millimolar absorptivity.

Plasma samples were analyzed for water‐soluble vitamins (B_3_ [niacin], B_5_ [pantothenic acid], B_12_ [cyanocobalamin], and C [ascorbic acid]). Plasma vitamin B_3_ and B_5_ samples were prepared according to the methods described by Dawson et al. (1988) and Aslam et al. (2008) and were analyzed by high‐performance liquid chromatography (HPLC) using a Waters HPLC instrument equipped with a Waters 717 Plus autosampler and Waters 510 pump. Plasma samples were diluted for analysis of vitamin C according to the methods described by Behrens and Madère (1987) and were also analyzed by HPLC. Plasma vitamin B_12_ levels were determined using a competitive binding radioimmunoassay kit (ICN, Costa Mesa, California), in which nonspecific vitamin B_12_ binding R‐proteins were removed by affinity chromatography.

### Fatty acids

Plasma samples were analyzed for fatty acids as described by Phillips et al. (2010). Samples were methylated as described in Parks and Goins (1994) and were reconstituted in hexane prior to analysis. Methyl esters were injected into a gas chromatograph (6890 Series II; Hewlett Packard, Avondale, Pennsylvania) fitted with a flame ionization detector and an automatic injector. Individual fatty acids were identified by comparing their retention times to those of known standards (12:0 and 27:0) that were added into each sample prior to methylation (Phillips et al. 2010). Fatty acid results are expressed as weight percentages of total fatty acids.

### Data analysis

Data were tested for normality using Anderson–Darling tests, and outliers were identified and removed according to criteria specific to Dixon's test and Tukey's test. Patterns between blood analytes and sex or life history state (juvenile or adult) were explored using nonparametric Wilcoxon rank‐sum tests. Correlations between blood analytes, longline soak times, and shark size were explored using linear regression. For life history comparisons, females larger than 220 cm TL and males larger than 190 cm TL were considered mature (Goldman et al. 2006).

Reference intervals encompass the central 95% of a healthy reference population. In nonnormally distributed (skewed) data, percentiles are used to establish RIs and the 2.5th and 97.5th percentiles are the upper and lower limits for the RIs (Friedrichs et al. 2012). We established RIs for each analyte by using nonparametric ranking methods (*N* ≤ 40) or by parametric/robust methods from native and Box–Cox transformed values (*N* > 40). The 90% confidence intervals around these limits were determined nonparametrically (*N* > 120) or through bootstrapping methods (*N* < 120) using Reference Value Advisor freeware (Geffré et al. 2009, 2011; Friedrichs et al. 2012). Statistical differences in blood analytes between sexes and/or life stages were identified, and separate RIs were calculated. All individuals included in the determination of RIs were presumed healthy based on physical appearance and external examination. Instances in which data were below the analyzer detection limit (e.g., censored data; <8% of total observations) were explored using the Kaplan–Meier estimator as a nonparametric maximum likelihood estimator (Helsel 2006). The median is the only descriptive statistic reported for censored data, and RIs were not determined for parameters that had more than 50% of their data censored. Values were considered to be significantly different at *P*‐values less than 0.05. Data analyses were performed with JMP version 10 (SAS Institute, Cary, North Carolina) and ProUCL version 5 (U.S. Environmental Protection Agency, Durham, North Carolina).

## Results

Blood samples were collected from 153 Sand Tigers ranging in size from 122 to 272 cm TL (101–233 cm FL) over the course of this study. Mean ± SD longline soak time was 2.9 ± 0.8 h, with a maximum soak time noted at 5.3 h for a single longline set. No differences were detected for any parameter based on year, and the data were pooled for subsequent analyses. The sex ratio of captured Sand Tigers was nearly equal (79 females and 74 males), and there was no significant difference in FL (*Z* = −0.127, *P* = 0.90) or TL (*Z* = −0.20, *P* = 0.84) between sexes. Of the 153 animals captured, 103 were later categorized as juveniles and 53 were categorized as adults. Average ± SD surface water temperature, salinity, and dissolved oxygen were 26.2 ± 1.8°C, 25.9 ± 2.1‰, and 5.3 ± 1.6 mg/L, respectively.

### Acid–Base and Blood Gases

Blood pH was near neutral in all individuals examined, whereas values of pO_2_, pCO_2_, HCO_3_, sO_2_, and lactate were highly variable (Table 1). Blood lactate values ranged from 2.3 to 18.1 mmol/L at capture. There were no significant differences between male and female sharks for any of the blood gas analytes (all *P* > 0.05). Juvenile sharks had significantly lower sO_2_ levels (47.7%) than adult animals (63.2%; χ^2^ = 4.49, df = 1, 113, *P* = 0.0340; Table 1). Reference intervals were not determined for blood gas analytes since they are impacted by capture and handling and would not represent baseline conditions.

**TABLE 1:** Descriptive statistics for postcapture blood gas parameters (temperature corrected) for Sand Tigers captured by longline (HCO_3_ = bicarbonate; pCO_2_ = partial pressure of carbon dioxide; pO_2_ = partial pressure of oxygen; sO_2_ = percent oxygen saturation).

Parameter	Mean	SD	Median	Min–Max	*n*
pH	7.2	0.1	7.2	7.0–7.4	124
pO_2_ (mm Hg)	30	28	20	5–138	124
pCO_2_ (mm Hg)	8.5	1.9	8.8	5.4–12.5	124
HCO_3_ (mmol/L)	3.9	0.9	3.9	1.8–5.7	124
sO_2_ (%)	50	37	48	3–99	124
Juveniles	48	39	43	3–99	78
Adults	63	30	67	7–99	46
Lactate (mmol/L)	8.8	3.3	8.5	2.3–18.1	124

### Hematology and Cell Counts

Hematocrit levels did not significantly differ between sexes (χ^2^ = 0.282, df = 1, 127, *P* = 0.5952), but a higher mean percentage of red blood cells was measured in juvenile animals (24%) compared with adult animals (22.4%; χ^2^ = 9.35, df = 1, 116, *P* = 0.0022; Table 2). Lymphocytes and CEGs were the most commonly identified WBCs, while monocytes and basophils were rarely observed (Table 2). Juvenile animals had significantly higher total WBCs (59.7 × 10^3^ cells/μL; χ^2^ = 9.96, df = 1, 51, *P* = 0.0016) and lymphocytes (41.1 × 10^3^ cells/μL; χ^2^ = 6.93, df = 1, 51, *P* = 0.0085) and significantly lower nonsegmented FEGs (0.7 × 10^3^ cells/μL; χ^2^ = 5.85, df = 1, 51, *P* = 0.0156) compared with adult sharks (43.0 × 10^3^, 22.2 × 10^3^, and 0.9 × 10^3^ cells/μL, respectively; Table 2).

**TABLE 2:** Descriptive statistics and reference intervals (RIs) with 90% confidence limits for plasma total solids and hematological cell counts in wild Sand Tigers. Descriptive statistics for analyte levels that were significantly different between males and females or between juveniles and adults are also presented under the associated parameter heading. Leukocyte differentials (diff) are presented both in absolute and relative form (CEGs = coarse eosinophilic granulocytes; FEG = fine eosinophilic granulocytes; G = Gaussian; LCL = lower 90% confidence limit; ND = not determined; NG = non‐Gaussian; ns = nonsegmented; PA = parametric method; PT = parametric method after Box–Cox transformation; RT = robust method after Box–Cox transformation; s = segmented; UCL = upper 90% confidence limit; WBCs = white blood cells).

Parameter	Diff % (±SD)	Mean	SD	Median	Min–Max	LCL	RI	UCL	*n*	Distribution	Method
Total solids (g/dL)		6.6	0.8	6.4	5.2–10.2	5.2–5.6	5.3–8.2	7.8–10.2	129	G	PA
Hematocrit (%)		23.5	2.4	24	18–30	18–20	18.2–28.0	27–30	127	NG	RT
Juveniles		24	2.2	24	18–30	18–20	19.3–29.5	27.8–30	89	NG	RT
Adults		22.4	2.5	22	18–27	16.5–18.8	17.5–28	26.4–29.5	28	NG	RT
Total WBCs (×10^3^ cells/μL)		52.9	17.8	52.3	15.5–92.6	15.4–23.8	16.5–90.7	84.2–92.6	53	G	PT
Juveniles		59.7	15.0	57.0	39.8–92.6	33.6–39.7	36.0–97.6	85.6–110.5	32	G	PT
Adults		43	16.7	40.5	15.5–72.4	ND	9.2–81.8	ND	19	G	PT
sCEGs (×10^3^ cells/μL)	11.5 ± 5.0	6.1	2.6	5.8	1.1–14.3	1.1–2.6	1.2–13.4	11.0–14.3	53	G	PT
nsCEGs (×10^3^ cells/μL)	3.5 ± 3.2	1.9	1.7	1.6	0.0–7.9	0.0	0.0–7.8	4.9–7.9	53	NG	RT
sFEGs (×10^3^ cells/μL)	4.5 ± 3.9	2.4	2.1	2.1	0.0–8.5	0.0–1.6	0.0–8.3	6.3–8.5	53	NG	RT
nsFEGs (×10^3^ cells/μL)	1.6 ± 1.9	0.8	1.0	0.5	0.0–4.2	0.0	0.0–3.7	2.6–4.2	52	NG	RT
Juveniles		0.7	1.0	0.3	0.0–4.2	ND	ND	ND	32	NG	
Adults		0.9	0.9	0.9	0.0–3.4	ND	ND	ND	21	NG	
Neutrophils (×10^3^ cells/μL)	11.1 ± 4.3	5.9	2.2	5.8	1.6–11.6	1.6–2.3	1.6–11.3	9.5–11.6	52	G	PT
Lymphocytes (×10^3^ cells/μL)	66.5 ± 9.4	35.2	5	35.7	25.3–47.1	25.4–28.2	25.8–45.9	42.8–47.1	52	G	PT
Juveniles		41.1	5.4	42.7	28.6–49.0	21.0–32.1	26.7–50.7	48.8–52.1	32	G	PT
Adults		27.2	3.8	26.7	22.8–38.3	20.9–22.9	21.7–39.3	33.6–47.8	21	G	PT
Monocytes (×10^3^ cells/μL)	1.2 ± 1.2	0.6	0.6	0.5	0.0–2.6	0.0	0.0–4.7	2.0–2.6	52	NG	RT
Basophils (×10^3^ cells/μL)	0.2 ± 0.5	0.1	0.3	0.0	0.0–1.6	0.0	0.0–2.3	0.5–1.6	52	NG	RT

### Biochemistry and Protein Fractions

Plasma biochemistry values for glucose were high (mean = 64 mg/dL; Table 3) compared to values measured in aquarium Sand Tigers (Anderson et al. 2012). Values for creatinine were below the level of analyzer detection (<0.2 mg/dL); therefore, RIs were not determined. No significant differences were detected between male and female Sand Tigers for any of the plasma biochemistry analytes (all *P* > 0.05). Plasma phosphorus (8.1 mg/dL; χ^2^ = 37.11, df = 1, 143, *P* < 0.0001), glucose (66 mg/dL; χ^2^ = 68.27, df = 1, 143, *P* = 0.009), amylase (831 IU/L; χ^2^ = 14.76, df = 1, 144, *P* = 0.0001), ALP (18 IU/L; χ^2^ = 36.81, df = 1, 141, *P* < 0.0001), and AST (28 IU/L; χ^2^ = 14.31, df = 1, 143, *P* = 0.0002) levels were significantly higher in juvenile animals compared to adult sharks (6.7 mg/dL, 60 mg/dL, 704 IU/L, 11 IU/L, and 21 IU/L, respectively; Table 3).

**TABLE 3:** Descriptive statistics and reference interval (RIs) with 90% confidence limits for plasma biochemistry values in wild Sand Tigers. Descriptive statistics for analyte levels that were significantly different between males and females or between juveniles and adults are also presented under the associated parameter heading (ALP = alkaline phosphatase; ALT = alanine aminotransferase; AST = aspartate aminotransferase; BUN = blood urea nitrogen; CHOL = cholesterol; CPK = creatinine phosphokinase; G = Gaussian; LCL = lower 90% confidence limit; mOsm = milliosmoles; ND = not determined; NG = non‐Gaussian; PA = parametric method; RT = robust method after Box–Cox transformation; UCL = upper 90% confidence limit).

Parameter	Mean	SD	Median	Min–Max	LCL	RI	UCL	*n*	Distribution	Method
BUN (mg/dL)	845	123	843	44–1,138	44–713	626–1,018	1,000–1,138	153	NG	RT
Creatinine (mg/dL)	ND	ND	0.2	ND	ND	ND	ND	153	NG	
Na (mmol/L)	254	16	255	200–294	200–224	212–283	278–294	152	NG	RT
K (mmol/L)	4.2	0.9	4.3	1.2–5.7	1.2–2.4	2.1–5.5	5.3–5.7	152	NG	RT
Cl (mmol/L)	230	14	232	172–258	172–200	185–256	252–258	152	NG	RT
Osmolarity (mOsm/L)	815	61	813	462–936	462–721	676–913	900–936	152	NG	RT
Ca (mg/dL)	15.1	0.8	15.1	12.4–17.1	12.4–13.6	13.6–16.7	16.4–17.1	153	G	PA
P (mg/dL)	7.7	1.3	7.8	4.2–12.2	4.2–5.5	5.2–10.4	9.7–12.2	152	G	PA
Juveniles	8.1	1.1	8.1	4.5–12.2	4.5–6.5	6.1–10.5	9.7–12.2	103	G	PA
Adults	6.7	1.4	6.5	4.2–11.6	4.2–5.3	4.2–11.6	9.5–11.6	40	G	PA
Mg (mg/dL)	2.9	0.3	2.9	0.4–3.9	0.4–2.6	2.5–3.6	3.3–3.9	152	NG	RT
Glucose (mg/dL)	64	12	64	36–97	36–44	40–90	88–97	152	G	PA
Juveniles	66	11	65	40–97	40–51	43–93	88–97	103	G	PA
Adults	60	13	60	36–90	36–38	36–90	79–90	40	G	PA
Amylase (IU/L)	796	171	789	41–1,422	41–572	523–1,160	1,069–1,422	153	NG	RT
Juveniles	831	167	817	504–1,422	504–596	543–1,232	1,108–1,422	103	NG	RT
Adults	704	161	712	41–987	41–552	76–987	925–987	42	NG	RT
ALP (IU/L)	16	6	16	5–40	5–7	5.8–32.2	27–40	150	NG	RT
Juveniles	18	6	17	6–40	6–9	7–33	28–40	103	NG	RT
Adults	11	4	9	5–22	3.7–5.3	4.3–22	19–25	39	NG	RT
ALT (IU/L)	8.5	4.2	8.0	3–22	3–3	3–17	15–22	120	G	PA
AST (IU/L)	26	14	22	6–89	6–11	10–77	53–89	151	NG	RT
Juveniles	28	15	24	10–89	10–12	11–81	53–89	102	NG	RT
Adults	21	13	17	6–76	6–10	6.2–74.8	41–76	42	NG	RT
CPK (IU/L)	105	144	56	15–832	15–19	16–645	448–832	150	NG	RT
CHOL (mg/dL)	55	13	53	23–90	23–37	28–82	80–90	152	G	PA

Plasma protein fraction 1 (0.16 g/dL) and fraction 2 (0.13 g/dL) were low compared to other fractions. The largest globulin fraction was fraction 4 (3.5 g/dL; Table 4). No significant differences in protein fractions were detected between males and females (all *P* > 0.05). Fraction 4 was significantly lower in juveniles (3.4 g/dL; χ^2^ = 7.66, df = 1, 35, *P* = 0.0057) than in adults (3.8 g/dL; Table 4), while fraction 3 levels were significantly higher in juveniles (1.6 g/dL; χ^2^ = 6.85, df = 1, 35, *P* = 0.0088) compared to adults (1.3 g/dL; Table 4). No other differences in protein fractions were detected between juveniles and adults (*P* > 0.05).

**TABLE 4:** Descriptive statistics and reference interval (RIs) with 90% confidence limits for plasma protein fractions in wild Sand Tigers. Descriptive statistics for analyte levels that were significantly different between males and females or between juveniles and adults are also presented under the associated parameter heading (G = Gaussian; LCL = lower 90% confidence limit; ND = not determined; PA = parametric method; UCL = upper 90% confidence limit).

Parameter	Mean	SD	Median	Min–Max	LCL	RI	UCL	*n*	Distribution	Method
Total protein (g/dL)	5.7	0.7	5.7	4.6–8.0	4.5–4.9	4.7–7.4	6.9–8.3	35	G	PA
Fraction 1 (g/dL)	0.16	0.09	0.11	0.05–0.40	0.03–0.05	0.04–0.54	0.31–0.90	35	G	PA
Fraction 2 (g/dL)	0.13	0.06	0.13	0.06–0.24	0.06–0.08	0.06–0.24	0.20–0.27	35	G	PA
Fraction 3 (g/dL)	1.5	0.3	1.5	1.0–2.1	0.8–1.1	0.9–2.1	1.9–2.2	35	G	PA
Juveniles	1.6	0.3	1.6	1.7–2.1	1.0–1.3	1.1–2.2	2.0–2.4	20	G	PA
Adults	1.3	0.3	1.4	1.0–1.7	ND	0.8–1.9	ND	15	G	PA
Fraction 4 (g/dL)	3.5	0.6	3.5	2.4–5.6	2.4–2.8	2.6–5.1	4.5–5.8	35	G	PA
Juveniles	3.4	0.7	3.2	2.4–5.6	2.3–2.7	2.5–5.4	4.1–ND	20	G	PA
Adults	3.8	0.4	3.7	2.9–4.8	ND	2.8–4.7	ND	15	G	PA
Fraction 5 (g/dL)	0.39	0.08	0.38	0.28–0.56	0.23–0.28	0.25–0.55	0.51–0.60	35	G	PA

### Trace Minerals and Heavy Metals

Trace mineral levels in Sand Tigers were similar between sexes for all minerals measured (all *P* > 0.05), with the exception of Se (Table 5). Significantly higher levels of both plasma Se (203 ng/mL; χ^2^ = 10.68, df = 1, 155, *P* = 0.0011) and whole‐blood Se (746 ng/mL; χ^2^ = 5.34, df = 1, 154, *P* = 0.0208) were noted in males compared to females (plasma Se: 186 ng/mL; whole‐blood Se: 675 ng/mL). Levels of Co, Mn, Zn, and As varied with life history stage (Table 5). Mean Co (2.1 ng/mL; χ^2^ = 9.56, df = 1, 143, *P* = 0.0020), Mn (15.7 ng/mL; χ^2^ = 6.98, df = 1, 143, *P* = 0.0082), and Zn (0.48 μg/mL; χ^2^ = 11.44, df = 1, 143, *P* = 0.0007) levels were significantly higher in juvenile sharks, whereas As levels (1,103 ng/mL; χ^2^ = 28.95, df = 1, 141, *P* < 0.0001) were significantly higher in adult sharks. A large portion of the analyzed values for Cd (71%), Pb (67%), Hg (77%), and Tl (71%) were below the level of analyzer detection (<0.50, <0.50, <0.25, and <0.50 ug/ml, respectively), and RIs were not determined.

**TABLE 5:** Descriptive statistics and reference interval (RIs) with 90% confidence limits for trace minerals, heavy metals, and vitamins in wild Sand Tigers. Descriptive statistics for analyte levels that were significantly different between males and females or between juveniles and adults are also presented under the associated parameter heading (G = Gaussian; LCL = lower 90% confidence limit; ND = not determined; NG = non‐Gaussian; P = plasma; PA = parametric method; PT = parametric method after Box–Cox transformation; RT = robust method after Box–Cox transformation; UCL = upper 90% confidence limit; W = whole blood).

Parameter	Mean	SD	Median	Min–Max	LCL	RI	UCL	*n*	Distribution	Method
As (ng/mL; W)	740	494	583	282–3,175	282–362	326–2,596	1,779–3,175	154	NG	RT
Juveniles	594	269	541	282–2,077	282–348	320–1,640	937–2,077	102	NG	RT
Adults	1,103	699	790	460–3,175	460–488	461–3,159	2,752–3,175	41	NG	RT
Cd (ng/mL; W)	ND	ND	5	ND	ND	ND	ND	154	NG	
Co (ng/mL; P)	1.96	0.64	1.96	0.19–4.28	0.19–1.01	0.67–3.28	3.09–4.28	155	G	PA
Juveniles	2.1	0.6	2.1	0.4–3.8	0.4–1.1	0.8–3.4	3.1–3.8	103	G	PA
Adults	1.8	0.7	1.7	0.20–4.28	0.20–0.99	0.2–4.2	3.0–4.3	41	G	PA
Cu (μg/mL; P)	0.38	0.09	0.38	0.02–0.60	0.02–0.23	0.13–0.51	0.49–0.60	155	G	PA
Fe (μg/dL; P)	17	8	15	10–62	10	10–42	31–62	125	G	PT
Pb (ng/mL; W)	ND	ND	5	ND	ND	ND	ND	154	NG	
Mn (ng/mL; P)	13.8	13.7	9.5	0.4–77.0	0.4–2.1	1.16–53.40	44.2–77.1	155	NG	RT
Juveniles	15.7	15.5	10.6	0.4–77.0	0.40–2.16	0.96–65.10	49.9–77.1	103	NG	RT
Adults	9.0	7.7	6.7	0.8–36.3	0.80–1.73	0.82–35.90	20.9–36.3	41	NG	RT
Hg (ng/mL; W)	ND	ND	25	ND	ND	ND	ND	154	NG	
Mo (ng/mL; P)	1.9	2.6	1.5	0.2–31.0	0.2–0.7	0.4–3.5	2.9–31.2	155	NG	RT
Se (ng/mL; P)	194	41	196	37–293	37–127	88–266	259–293	154	NG	RT
Males	203	41	208	67–275	67–127	89–266	259–275	76	NG	RT
Females	186	39	190	37–293	37–129	52–270	254–293	78	NG	RT
Se (ng/mL; W)	709	181	685	217–1,660	217–467	433–1,087	1,061–1,660	154	NG	RT
Males	746	205	721	217–1,660	217–509	424–1,287	1,069–1,660	75	NG	RT
Females	675	148	673	400–1,078	400–460	409–1,061	937–1,078	79	NG	RT
Tl (ng/mL; W)	ND	ND	5	ND	ND	ND	ND	154	NG	
Zn (μg/mL; P)	0.46	0.11	0.47	0.07–0.76	0.07–0.28	0.22–0.67	0.63–0.76	155	G	PA
Juveniles	0.48	0.11	0.48	0.07–0.76	0.07–0.29	0.21–0.69	0.63–0.76	103	G	PA
Adults	0.42	0.11	0.41	0.12–0.76	0.12–0.28	0.13–0.76	0.54–0.76	41	G	PA
Vitamin A (ng/mL; P)	69	28	64	22–156	22–35	27–144	131–156	143	NG	RT
Vitamin B_3_ (μg/L; P)	37	13	36	17–59	17–19	17–59	58–59	99	NG	RT
Vitamin B_5_ (μg/L; P)	284	73	297	153–404	153–157	155–398	387–404	99	NG	RT
Vitamin B_12_ (ng/L; P)	73	8	74	58–86	58–60	58–85	84–86	99	NG	RT
Vitamin C (mg/dL; P)	0.48	0.17	0.48	0.07–0.73	0.07–0.20	0.18–0.73	0.72–0.73	100	NG	RT
Vitamin E (μg/mL; P)	1.15	0.5	1.13	0.22–2.67	0.22–0.44	0.38–2.29	2.06–2.67	155	G	PT
Juveniles	1.19	0.48	1.16	0.22–2.67	0.22–0.45	0.36–2.26	2.06–2.67	103	G	PT
Adults	1.03	0.56	0.80	0.34–2.51	0.34–0.44	0.34–2.50	1.94–2.51	41	G	PT

### Vitamins

No differences in vitamin levels were detected between male and female Sand Tigers (all *P* > 0.05); however, differences between life history stages were noted for vitamin E (Table 5). Mean vitamin E levels were significantly higher in juveniles (1.19 μg/mL; χ^2^ = 4.93, df = 1, 143, *P* = 0.0264) compared to adults (1.03 μg/mL).

### Reproductive Hormones

Plasma hormone levels varied based on maturity stage and sex. Significantly higher levels of estradiol were measured in females (both juveniles and adults) than in males (both juveniles and adults; χ^2^ = 28.22, df = 1, 97, *P* < 0.0001), whereas significantly higher levels of both progesterone (χ^2^ = 14.11, df = 1, 98, *P* = 0.0002) and testosterone (χ^2^ = 44.47, df = 1, 90, *P* < 0.0001) were observed in males compared to females (Table 6). Juvenile males had significantly lower mean testosterone levels (4.7 ng/mL; χ^2^ = 8.38, df = 1, 67, *P* = 0.0038) than adult males (6.6 ng/mL). No other hormonal differences were observed between adult and juvenile sharks (all *P* > 0.05). Reference intervals were not determined for plasma hormone levels because those values would vary due to the seasonal reproductive cycle of the species.

**TABLE 6:** Descriptive statistics for plasma hormone levels in juvenile and adult wild Sand Tigers.

	Males					Females				
Parameter	Mean	SD	Median	Min–Max	*n*	Mean	SD	Median	Min–Max	*n*
**Juveniles**										
Estradiol (pg/mL)	125.7	18.0	125.9	92.9–149.6	18	478.3	336.9	465	92.5–1,340.2	56
Progesterone (ng/mL)	0.28	0.1	0.29	0.10–0.45	18	0.19	0.11	0.18	0.05–0.65	56
Testosterone (ng/mL)	4.7	1.8	4.4	1.8–11.4	40	0.65	0.15	0.66	0.37–0.85	13
**Adults**										
Estradiol (pg/mL)	134.8	38.0	142.4	93.6–168.4	3	777.1	191	858.8	505.8–1,000.0	11
Progesterone (ng/mL)	0.29	0.17	0.24	0.15–0.48	3	0.17	0.13	0.11	0.05–0.48	11
Testosterone (ng/mL)	6.6	3.0	6.2	2.6–16.0	28					

### Fatty Acids

Twenty‐six individual fatty acids were detected in Sand Tiger plasma (Table 7). Polyunsaturated fatty acids (PUFAs; 36.0%), saturated fatty acids (31.5%), and monounsaturated fatty acids (29.5%) were observed in nearly equal proportions in plasma. Dominant fatty acids (>5%) in decreasing order of relative importance included palmitic acid (16:0; 17.5%), oleic acid (18:1[n‐9], where the number to the left of the colon is the number of carbon atoms, the number to the right of the colon is the number of double bonds, and the number after the hyphen indicates the position of the first double bond from the methyl end; 15.9%), docosahexaenoic acid (DHA; 22:6[n‐3]; 11.7%), stearic acid (18:0; 11%), eicosapentaenoic acid (EPA; 20:5[n‐3]; 8.2%), and arachidonic acid (20:4[n‐6]; 5.9%). The ratio of n‐3/n‐6 PUFAs was 2.5. The only significant difference between sexes was for a single fatty acid, EPA, with male sharks having significantly higher levels of EPA (8.4%; χ^2^ = 5.82, df = 1, 99, *P* = 0.0159) than females (8.0%). No differences in fatty acids were detected based on maturity (all *P* > 0.05).

**TABLE 7:** Descriptive statistics for plasma fatty acids in wild Sand Tigers (AA = arachidonic acid; ALA = alpha linolenic acid; DHA = docosahexaenoic acid; EPA = eicosapentaenoic acid; MUFAs = monounsaturated fatty acids; PUFAs = polyunsaturated fatty acids; SFAs = saturated fatty acids).

Fatty acid	Mean	SD	Median	Min–Max	*n*
14:0 (%)	0.17	0.24	0.01	0.00–0.75	99
15:0 (%)	0.81	0.68	0.36	0.26–2.96	99
16:0 (%)	17.5	2.80	18.4	13.25–22.42	99
16:1 (%)	3.50	0.52	3.5	1.73–4.50	99
16:1 trans‐9 (%)	0.43	0.05	0.41	0.22–0.52	99
17:0 (%)	1.04	0.18	1.0	0.16–1.32	99
17:1(n‐7) (%)	0.43	0.06	0.41	0.22–0.61	99
18:0 (%)	11.0	1.17	11.1	7.0–15.4	99
18:1 (%)	4.86	0.72	4.65	3.75–6.84	99
18:1(n‐9) (%)	15.90	2.00	16	8.45–20.20	99
18:2(n‐6) (LA; %)	2.44	0.50	2.2	1.39–4.01	98
18:3(n‐3) (ALA; %)	0.57	0.11	0.52	0.31–0.87	98
20:0 (%)	0.90	0.13	0.83	0.44–1.12	93
20:1(n‐9) (%)	1.23	0.16	1.14	0.80–1.61	98
20:1(n‐15) (%)	0.56	0.03	0.56	0.49–0.60	12
20:2(n‐6) (%)	0.44	0.06	0.4	0.24–0.62	92
20:3(n‐3) (%)	0.71	0.09	0.68	0.37–0.98	97
20:3(n‐6) (%)	0.49	0.06	0.47	0.40–0.69	92
20:4(n‐6) (AA; %)	5.89	0.67	5.64	4.60–7.16	99
20:5(n‐3) (EPA; %)	8.20	0.94	8.34	5.25–12.28	99
Males	8.44	1.00	8.4	6.93–12.28	50
Females	8.00	0.83	8.0	5.25–10.72	49
22:1(n‐9) (%)	0.48	0.16	0.5	0.37–0.59	2
22:3(n‐3) (%)	0.63	0.08	0.59	0.49–0.78	99
22:4(n‐6) (%)	1.26	0.17	1.26	0.66–1.94	98
24:1(n‐9) (%)	1.22	0.15	1.16	0.57–1.52	98
22:5(n‐3) (%)	3.17	0.38	3.02	1.89–3.92	99
22:6(n‐3) (DHA; %)	11.74	1.93	11.75	4.21–18.70	99
Total SFAs (%)	31.5				
Total MUFAs (%)	29.5				
Total PUFAs (%)	36.0				
n‐3 polyenes (%)	24.9				
n‐6 polyenes (%)	10.0				
n‐3/n‐6 (%)	2.5				

## Discussion

This study provides comprehensive hematological RIs for a large number of wild Sand Tigers representing both sexes, a range of sizes, and immature and mature reproductive stages from Delaware Bay. Baseline blood values from wild, healthy Sand Tigers are necessary to inform decisions on veterinary and nutritional health, to optimize diet and vitamin supplementation, and to enhance reproductive success in aquarium animals. Blood values can be subject to alteration due to physiological effects of capture and handling‐related activities, and it is important to view sensitive blood parameters in light of these potentially altering events (Ahmed et al. 2020). Longline capture of Sand Tigers in this study produced a pronounced physiological stress response resulting in a mixed metabolic and respiratory acidosis (low pH, high lactate, and elevated pCO_2_). Sharks exposed to acute capture stress typically experience full physiological recovery within 12–24 h of release (Frick et al. 2012; Kneebone et al. 2013; Kilfoil et al. 2017).

A complete blood count, including hematocrit, provides information about the types, numbers, and morphology of cells in the blood, which can be used to evaluate animal health and to identify issues such as anemia, dehydration, infection, and trauma. Increases in hematocrit may be induced by a stress event as a result of red blood cell swelling associated with osmotic upset (Brill et al. 2008). Hematocrit levels (mean = 23.5%) measured in this study were comparable to those measured in aquarium Sand Tigers (24–26%) that were routinely handled for sampling (Stoskopf 2010; Anderson et al. 2012). Total WBC counts in this study (52.3 × 10^3^ cells/μL) were higher than counts previously measured in wild Sand Tigers (16 × 10^3^ cells/μL; Stoskopf 2010). Elevated WBC counts are typically indicative of stress, inflammation, parasite burden, and/or disease, but comparison of WBC counts among elasmobranchs is challenging due to inconsistencies in methodology and a lack of standardization in cell type identification.

Knowledge on the functions of elasmobranch WBC types is limited, and not all elasmobranch granulocytes have a mammalian counterpart (Arnold 2005). Based on the length of time for which Sand Tigers would have been on a hook in this study and the slow leukocyte response to stress in fishes (e.g., 12–24 h in Channel Catfish *Ictalurus punctatus*; Davis et al. 2008), it is unlikely that the high total WBC counts we observed would be related to the stress of capture. Furthermore, the leukocyte differentials we observed, including a predominance of lymphocytes, were within the normal ranges found in other species of sharks, both in aquariums and the wild (Arnold 2005; Stoskopf 2010; Haman et al. 2012). An increased ratio of granulocytes (neutrophils and/or heterophils depending on the taxon) to lymphocytes in a differential is generally regarded as a better indicator of stress than are total WBC counts (Davis et al. 2008). Granulocytosis (increased neutrophils and heterophils) and lymphopenia (decreased lymphocytes), which can indicate changes in immune strategy (Davis et al. 2008), were not observed in the leukocyte differentials in this study. The most likely explanation for the observed high WBC counts is that granulated thrombocytes were included in the absolute WBC counts even though they are not included in the differential. It is unclear whether the delayed time to blood processing had any significant effect on total WBC counts. Given the uncertainty surrounding the cause of high total WBC counts, the RIs should be viewed with caution.

Standard veterinary biochemical panels typically allow for the evaluation of disease across numerous organ systems using well‐defined, taxon‐specific reference ranges. However, little is known about the origin and physiological function in elasmobranchs for many of the biochemical parameters measured in a standard mammalian panel. Plasma biochemistry values in our study were comparable to serum values reported for wild Sand Tigers captured in Australian waters (Otway 2015) and for healthy Sand Tigers in aquaria (Anderson et al. 2012). In our study, plasma electrolyte levels (Na^+^, K^+^, and Cl^−^), which can be influenced by physiological capture stress in sharks (Brill et al. 2008; Frick et al. 2009, 2010), were comparable to values reported for both wild and aquarium Sand Tigers (Anderson et al. 2012; Otway 2015). This suggests that different capture techniques among studies resulted in similar minimal impacts on electrolyte balance for Sand Tigers. Osmolarity levels in our study (815 mmol/L) were lower than values reported for wild Sand Tigers (1,082 mmol/L; Otway 2015), although it should be noted that analytical methodologies were different between the two studies. Sodium and chloride concentrations contribute most significantly to the ion portion of plasma osmolarity, and the slightly lower Cl^−^ levels in our study likely explain the lower osmolarity levels compared to those reported by Otway (2015).

Urea synthesis in elasmobranchs occurs in the liver, and the kidneys regulate blood urea levels, with about 95% reabsorption occurring within renal tubules (Ballantyne 1997). The mean BUN value obtained in our study (845 mg/dL) was lower than the level reported by Otway (2015; 1,056 mg/dL). Conversely, TP levels measured in Sand Tigers in our study (5.7 g/dL) were higher than levels reported for wild Sand Tigers (3.0 g/dL; Otway 2015) and healthy aquarium animals (3.68 g/dL [unknown method]; Anderson et al. 2012). Although direct comparison of TP values among studies must be made with caution due to potential differences in analytical methods, BUN and TP can be valuable indicators of feeding status in sharks, as increases in TP and decreases in the BUN : TP ratio are associated with increased feeding rates in sharks (Wyatt et al. 2019).

Sand Tigers in our study exhibited higher mean glucose levels (64 mg/dL) than were reported for Sand Tigers captured by SCUBA divers (48.7 mg/dL; Otway 2015) and for aquarium Sand Tigers routinely handled for blood sampling (34 mg/dL; Anderson et al. 2012). Elevated glucose levels may be related to feeding status but are more likely indicative of a physiological stress response. Glucose levels may increase under conditions of prolonged stress in elasmobranchs (Skomal and Bernal 2010), and the onset of glucose mobilization appears to occur prior to the accumulation of lactate in the blood (Wells et al. 1986). Given the potential for capture and handling to elevate glucose levels in elasmobranchs, the RIs for glucose should also be interpreted with caution.

Protein electrophoresis has been widely used in human and veterinary medicine to evaluate albumin and globulin fractions (α‐, β‐, and γ‐globulins) for assessment of ongoing inflammatory processes like infectious disease, parasitism, trauma, and/or poor nutritional plane (Cray et al. 2015). Given the unique physiology of elasmobranchs, there is interest in applying this technique for the detection of inflammatory response in managed elasmobranchs since classic plasma biochemistry and hematology testing alone may not be optimal for diagnosing disease in these species (Hyatt et al. 2016). In our study, fraction 1 was small (0.16 g/dL) and consistent with negligible levels of albumin previously reported for other elasmobranchs (Metcalf and Gemmell 2005; Cray et al. 2015; Hyatt et al. 2016). Fraction 2, which shares the migration characteristics of α‐1‐globulins in other taxa, was low (0.13 g/dL) and also was the least distinct fraction in this study and others (Cray et al. 2015; Hyatt et al. 2016). It is unknown which proteins comprise fraction 3 in elasmobranchs; however, this fraction in mammals, birds, and reptiles comprises the following α‐2‐globulins: α‐2‐macroglobulin, haptoglobin, and ceruloplasmin. Fraction 3 in Sand Tigers in our study (1.5 g/dL) was higher than the level measured in Cownose Rays *Rhinoptera bonasus* (0.72 g/dL; Cray et al. 2015) but was not as high as the level measured in Bonnetheads *Sphyrna tiburo* (2.28 g/dL; Hyatt et al. 2016), possibly reflecting species‐specific differences in lipoprotein characteristics (Hyatt et al. 2016). Fraction 4, similar to β‐globulin migrating fraction, was identified as the largest protein fraction in Sand Tigers (3.5 g/dL), similar to what has been noted in other elasmobranchs (Cray et al. 2015; Hyatt et al. 2016), and is believed to be correlated with very low‐density and low‐density lipoprotein cholesterol fractions (Cray et al. 2015). Lastly, fraction 5 is thought to represent γ‐globulins, specifically immunoglobulin M, the primary immunoglobulin found in elasmobranchs (Cray et al. 2015).

The protein fractions determined in this study provide novel baseline information for apparently healthy Sand Tigers, which can be useful for evaluating the health and disease state of aquarium cohorts. For example, Hyatt et al. (2016) found significant decreases in fraction 3 and increases in fraction 5 of clinically abnormal Bonnetheads (e.g., those with bacterial or fungal infections, skin lesions, or wound healing) compared with healthy individuals, suggesting that this diagnostic technique has great promise for detecting immunological stimulation in response to subclinical inflammatory processes. This is significant given the high incidence of spinal trauma and resulting spinal deformity noted in aquarium Sand Tiger populations (Anderson et al. 2012).

Trace minerals and vitamins are micronutrients required for normal growth, reproduction, health, and maintenance of fish metabolism (NRC 2011). Plasma and whole‐blood trace mineral and vitamin levels measured in our study are the first reported for this species in the wild. These baselines are valuable for assessing the nutritional health of aquarium cohorts since trace mineral and vitamin levels in fish are largely acquired from the diet (Watanabe et al. 1997). Limited mineral (Zn) and vitamin (A, C, and E) values have been reported in aquarium Sand Tigers, as they relate to the high prevalence of spinal deformity in this species (Anderson et al. 2012). Mean Zn levels measured in wild Sand Tigers in our study (0.46 μg/mL) were markedly lower than levels reported for healthy sharks in aquaria (mean = 7.89 μg/mL) and were lower than levels in aquarium sharks affected with spinal deformities (mean = 4.12 μg/mL). Similarly, vitamin A (69 ng/mL), vitamin C (0.48 mg/dL), and vitamin E (1.15 μg/mL) levels measured in our study were lower than values reported for healthy (mean = 18,750 ng/mL, 0.62 mg/dL, and 7.89 μg/mL, respectively) and affected (mean = 14,070 ng/mL, 0.41 mg/dL, and 4.12 μg/mL, respectively) aquarium animals (Anderson et al. 2012). The vitamin and mineral differences between wild and aquarium Sand Tigers are largely attributed to the higher levels in aquarium animals due to vitamin supplementation of the diet, and this is particularly evident in the vitamin A and vitamin E levels reported for managed Sand Tigers. The lower circulating levels of these vitamins and minerals in wild Sand Tigers suggest that nutrition may play a lesser and/or secondary role (to capture‐ and transport‐induced trauma) in the spinal deformities seen in managed populations (Anderson et al. 2012). Additionally, data from this study provide baseline information on circulating vitamin levels necessary for evaluating vitamin supplementation in aquarium populations, where over‐ and undersupplementation of fat‐soluble vitamins can be an issue.

### Variation with Maturity

Differences in blood analytes were detected with Sand Tiger maturity status (based on TL). Notably, higher values for hematocrit, total WBC counts, lymphocytes, and glucose were measured in juvenile sharks compared with adults. Juvenile teleosts have been shown to exhibit higher counts of both total leukocytes and lymphocytes than adults, which may reflect an immune system that is not fully developed (Ahmed et al. 2020). However, taken together, these elevated values may also indicate that juveniles are more prone to the effects of capture stress than adult Sand Tigers.

We also observed differences in a number of plasma biochemistry values (phosphorus, amylase, AST, and ALP) between juvenile and adult Sand Tigers in our study. Otway (2015) recorded no variation in serum biochemistry analytes among Sand Tigers of different sizes, ages, and sexes, with the exception of increased ALP levels with length and age in female sharks, although the sample size in that study was considerably smaller (*N* = 30) than our sample size. The significance of variable plasma biochemistry values in our study is not clear, as variations in diet and age may influence phosphorus levels (NRC 2011). Amylase is a starch‐digesting enzyme, and while fish have the same carbohydrate metabolic pathways as mammals, the value of monitoring plasma amylase for the purposes of detecting digestive disorders in elasmobranchs is unknown. Similarly, enzymes that are traditionally considered useful in serum and plasma biochemistry panels for evaluating potential organ damage in mammals (e.g., ALP, ALT, AST, and CPK) are difficult to interpret for elasmobranchs given the lack of information about the functional significance and tissue specificity of each enzyme (Anderson et al. 2010; E. O. Clarke and coworkers, unpublished abstract, International Association for Aquatic Animal Medicine conference, 2012). In mammals, AST is found in all tissues (except bone), with the highest concentrations in liver and skeletal muscle. However, in Red Lionfish *Pterois volitans*, AST enzyme activity is primarily detected in liver and heart tissue (Anderson et al. 2010), suggesting differences in the diagnostic significance of plasma AST levels across taxa. Further research on the diagnostic significance of these biochemistry parameters in elasmobranchs is needed. Differences between juvenile and adult Sand Tigers were detected for both trace minerals (Co, Mn, and Zn) and vitamin E. The slight differences observed among Sand Tigers in our study may reflect differences in feeding state (fed versus fasted), diet, and/or environment.

### Application to Breeding Programs

Differences in blood analytes between sexes may offer insight into physiological processes related to reproduction. Sand Tigers have among the lowest reproductive rates known for sharks (two pups every 2 years; Gilmore et al. 1983). This aplacental viviparous species is unique in that larger embryos feed on smaller embryos (intrauterine cannibalism) and then grow by feeding on unfertilized oocytes supplied by the mother, resulting in a single embryo born per uterine horn (Gilmore et al. 1983; Lucifora et al. 2002). Despite the large numbers of Sand Tigers in aquaria worldwide, successful reproduction has only occurred at four institutions to date (Henningsen et al. 2015).

Estradiol, progesterone, and testosterone levels have not been previously measured in wild Sand Tigers, and most of what is known about the reproductive endocrinology of Sand Tigers comes from animals that are maintained in aquaria. Estradiol concentrations in immature (mean = 478 pg/mL) and mature (mean = 777 pg/mL) female Sand Tigers in this study were similar to levels measured in serially sampled aquarium females that were identified as immature (<700 pg/mL) and mature (600–2,000 pg/mL; Rasmussen and Murru 1992). The lack of reproductive hormone profile differences between mature animals suggests that they were not reproductively active during the month of August, when samples were collected in this study. Reproductive hormone cycles of male and female Sand Tigers in aquaria suggest that summer months represent a lull in reproductive activity, as the lowest levels of steroid hormones were measured with the least amount of variation and low motility was detected in sperm during this period (Henningsen et al. 2008; Wyffels et al. 2020). Those results also suggest that temporal patterns of the reproductive cycle may be conserved in aquaria. While fisheries data provide some information on the seasonality of reproduction in Sand Tigers in the northwest Atlantic (Gilmore et al. 1983), research focusing on hormonal blood sampling in conjunction with reproductive ultrasound evaluation throughout the year is needed to understand the complete reproductive cycle and timing in this species.

Among the trace minerals, only Se was different between sexes, with male Sand Tigers having significantly higher plasma (203 ng/mL) and whole‐blood (746 ng/mL) levels of Se compared with females (186 and 675 ng/mL, respectively). Selenium is an essential trace mineral and an integral component of glutathione peroxidase (1.11.1.9), which, in conjunction with vitamin E, protects cells against oxidative damage and prevents nutritional muscular myopathy. As a result, Se compounds are capable of protecting tissues from the toxicity of heavy metals such as Cd and Hg (Watanabe et al. 1997). Dietary Se also plays a vital role in mammalian male reproduction. Male reproductive organ morphology, spermatogenesis, semen quality and motility, and fertility have been shown to be affected by excesses or deficiencies of Se in mammals (reviewed by Ahsan et al. 2014). Persky et al. (2012) also noted significantly higher Se levels in aquarium male Smooth Dogfish *Mustelus canis* (347 ng/mL) compared with females (230 ng/mL), although at lower circulating levels than measured in this study. The role of Se in fish reproduction is less well understood and limited to work with teleosts (Ogle and Knight 1989; Penglase et al. 2014). Further research is needed to understand the potential role of Se in shark reproduction, especially considering the lack of successful breeding of managed Sand Tigers.

Fatty acid monitoring is important for animal health since fish lack the de novo ability to biosynthesize long‐chain PUFAs, thus relying on their diet to obtain these essential fatty acids (Sargent et al. 1999; Tocher 2003). Deficiencies in essential fatty acids can result in fish pathologies, including reduced growth, myocarditis, fatty liver, fin erosions, lordosis, reduced reproductive potential, and mortality (NRC 2011). More specifically, dietary fatty acids have proven to be very important in the reproduction of several teleost fish species since they determine gonad composition—affecting not only sperm and egg quality and maturation (Izquierdo et al. 2001; Rodriguez‐Barreto et al. 2014; Zupa et al. 2017), but also the synthesis of eicosanoids that are autocrine mediators in the reproductive process (Sorbera et al. 2001; Tocher 2003).

The individual fatty acids comprising the bulk of the plasma fatty acids in Sand Tigers in our study included palmitic acid (16:0), stearic acid (18:0), and oleic acid (18:1[n‐9]), which are preferentially oxidized in fish (Tocher 2003). Differences in a single fatty acid, EPA (20:5[n‐3]), were detected between male and female Sand Tigers in our study. Although it is not known whether the incorporation of the n‐3 fatty acids DHA and EPA into plasma fractions differs between sexes in elasmobranchs, such differences between sexes have been documented in mammals. Males and females differ in their ability to synthesize EPA and DHA from their fatty acid precursor, alpha‐linolenic acid, and this has been linked with circulating levels of sex hormones (Huang and Horrobin 1987; Childs et al. 2008; Walker et al. 2014). In teleosts, the 20‐carbon PUFAs play an important structural role in sperm phospholipids and in ovarian physiology. Arachidonic acid (20:4[n‐6]) and EPA are precursors of eicosanoids, among which prostaglandins play an important role in vertebrate reproductive function (Stacey and Goetz 1982; Sorbera et al. 2001). The fatty acid composition of fish tissue reflects dietary lipid to a great extent (Henderson and Tocher 1987; Sargent et al. 1989), thus making it easy to manipulate the fatty acid composition of key reproductive tissues by altering dietary inputs. This has direct application to enhancing the reproductive efforts of Sand Tigers in aquaria by comparing the fatty acid composition of wild and aquarium‐reared sharks, similar to what has previously been studied in the aquaculture of commercially important teleost species (e.g., Zupa et al. 2017).

### Health of Delaware Bay

Sand Tigers can be predictably found in the waters of Delaware Bay during the summer months (June–August) before migrating south (Teter et al. 2015). Inorganic elements in fish are required in trace quantities for normal physiological function (NRC 2011). However, tissue concentrations can also be an indication of the level of environmental pollution, with tissue levels being influenced by both abiotic and biotic factors and the diet being the primary route of exposure. Trace mineral levels in fish are typically measured in liver and/or muscle tissue as a long‐term indication of exposure, while blood values typically depict short‐term exposure. Whole‐blood levels of Cd (<5 ng/mL), Pb (<5 ng/mL), and Hg (<25 ng/mL) were low in Sand Tigers in our study compared to levels of Cd, Pb, and Hg reported in Spiny Dogfish *Squalus acanthias* (200, 900, and 300 ng/mL, respectively) from the North Pacific (Haman et al. 2012). Conversely, mean blood As levels in Sand Tigers from Delaware Bay (740 ng/mL; present study) were lower than levels detected in Atlantic Sharpnose Sharks *Rhizoprionodon terraenovae* and Bonnetheads (3,100–3,500 ng/mL) sampled off the Georgia coast (Haman et al. 2012). While differences in blood fraction, foraging ecology, and environmental exposure make direct comparisons difficult, whole‐blood metal values measured in this study provide a snapshot of potential low environmental exposure for this migratory species during their time in Delaware Bay.

## Conclusions

This study provides RIs for a comprehensive suite of blood analytes measured in wild male and female Sand Tigers representing a range of age‐classes and size‐classes. This information will improve the evaluation of nutritional status and overall health of Sand Tigers held in aquariums, especially with respect to the common problem of spinal deformity in this population of animals. Values obtained in our study provide a reference for comparison with other populations of wild Sand Tigers, some of which have experienced drastic declines or are extremely vulnerable to fishing pressure (Carlson et al. 2009; Pollard and Smith 2009). Additionally, the wide range of parameters obtained for wild Sand Tigers in our study greatly advances understanding of the biology of these animals and provides information that is likely to improve breeding success for Sand Tigers in aquaria. Values obtained for sexually mature, gestating females in future studies would be especially beneficial for enhanced efforts toward the breeding of Sand Tigers.
